# Impact of general versus epidural anesthesia on early post-operative cognitive dysfunction following hip and knee surgery

**DOI:** 10.4103/0974-2700.76829

**Published:** 2011

**Authors:** Sripurna Mandal, Mina Basu, Jyotirmay Kirtania, Debabrata Sarbapalli, Ranabir Pal, Sumit Kar, Kanak Kanti Kundu, Ujjal Sarkar, Sampa Dutta Gupta

**Affiliations:** Department of Anesthesiology, India; 1 Department of Gastrointestinal and Hepatic Anesthesiology, School of Digestive and Liver Diseases, Institute of Postgraduate Medical Education and Research, Kolkata, India; 2 Department of Anesthesiology, Burdwan Medical College, West Bengal, India; 3 Department of Community Medicine, Sikkim Manipal Institute of Medical Sciences and Central Referral Hospital, 5^th^ Mile, Tadong, Gangtok, Sikkim-737102, India

**Keywords:** Anesthesia, cognitive dysfunction, epidural, general

## Abstract

**Background:**

Post-operative cognitive dysfunction is the subtle cerebral complication temporally seen following surgery. The aim of this study was to compare the influence of either general anesthesia (GA) or epidural anesthesia (EA) on the early post-operative neurocognitive outcome in elderly (>59 years) subjects undergoing hip and knee surgery.

**Methods:**

A total of 60 patients were recruited in a prospective, randomized, parallel-group study, comparable by age and sex. They were enrolled and randomized to receive either EA (*n* = 30) or GA (*n* = 30). All of them were screened using the Mini Mental State Examination (MMSE), with components of the Kolkata Cognitive Screening Battery. The operated patients were re-evaluated 1 week after surgery using the same scale. The data collected were analyzed to assess statistical significance.

**Results:**

We observed no statistical difference in cognitive behavior in either group pre-operatively, which were comparable with respect to age, sex and type of surgery. Grossly, a significant difference was seen between the two groups with respect to the perioperative changes in verbal fluency for categories and MMSE scores. However, these differences were not significant after the application of the Bonferroni correction for multiple analyses, except the significant differences observed only in the MMSE scores.

**Conclusions:**

We observed a difference in cognitive outcome with GA compared with EA. Certain aspects of the cognition were affected to a greater extent in this group of patients undergoing hip and knee surgery.

## INTRODUCTION

Cerebral insults that follow surgery are now clearly defined and categorized into distinct syndromes of post-operative delirium, cognitive dysfunction and major neurological complications like stroke. To what extent surgery or the anesthetic drugs are attributable for such consequences is difficult to determine as both are inseparable.[1] Cognitive dysfunction is an impairment of the information-processing abilities of the brain, like attention, perception, verbal abilities, learning and memory and abstract thinking.[2] Post-operative cognitive dysfunction (POCD) is one such subtle cerebral complication that is temporally seen following surgery. It is defined as decline of 20% or more in the cognitive screening scores on at least 20% or more of the psychometric tests performed pre-operatively and 1 week (early) and 3 months (delayed) post-operatively.[3] Alternatively, deterioration by some criterion number of standard deviation units can be used for its diagnosis. The aged brain is different from the younger brain in several important aspects, including size, distribution and type of neurotransmitters, metabolic functions and capacity for plasticity. For these reasons, the elderly brain is more vulnerable and susceptible to the adverse neurological sequelae of surgery and anesthesia.[4] Bedford in 1955 first published that anesthesia could have long-lasting amnesic effects. But, the term POCD has appeared only recently, covering a wide range of neuropsychological deviations, ranging from concentration impairment to delirium.[5] POCD is a condition definedas an abnormality on neuropsychological testing, which can afflict up to 14% of the older than 70 years patients undergoing electivesurgery with no convincingly demonstrated surgical, anesthetic andenvironmental risk factors.[6,7,8]

POCD after coronary artery bypass graft surgery was depicted to occur in 53% of the patients at discharge, 36% at 6 weeks and 24% at 6 months.[9] The description of POCD remained contentious and many diverse definitions were put forth in surgical literatures.[10,11] According to the Diagnostic and Statistical Manual of Mental Disorder, POCD is considered a mild neurocognitive disorder if the patient scores 12 or more points on the Delirium Rating Scale.[12]

Risk factors of POCD can be divided into age and co-morbidity dependent and those related to anesthesia and surgery. Cardiovascular, orthopedic and urologic interventions carry a higher risk of POCD. Orthopedic surgeries, particularly those on the hip and knee, are associated with a high incidence of acute POCD as studied by Koch *et al*.[13] Age is a well-established risk factor for the development of both early and delayed POCD, as demonstrated in ISPOCD1.[14,15]

Increasing age and duration of anesthesia, little education, a second operation, post-operative infection and respiratory complications are the risk factors for early POCD at 1 week but only age was a risk factor for late POCD at 3 months.[18] Acute post-operative pain has also been associated with poorer post-operative cognitive function. The degree of chronic pre-operative pain is however not related to pre-operative cognitive test performance.[17] History of alcohol abuse in elderly patients has been found to be a risk for post-operative cognitive decline in the domains of visuospatial abilities and executive functions.[18]

To what extent anesthesia itself is a causal factor for such deterioration of cognitive performance might be determined by a comparison of the post-operative decline of scores between groups receiving general anesthesia (GA) and regional anesthesia, respectively. In this context, an effort has been made in the present study to compare the cognitive changes in elderly patients undergoing hip and knee surgery following GA and epidural anesthesia (EA), respectively.

## METHODS

The study was undertaken in a tertiary care hospital of Kolkata during March 2007 and August 2008. It was a prospective, randomized, parallel-group studyrecruiting60 adults of the American Society of Anesthesiologists (ASA) physical status I and II, scheduled for hip and knee orthopedic surgery. For the purpose of sample size calculation, the Mini Mental State Examination (MMSE) component of the Kolkata Cognitive Screening Battery was used. In order to detect a difference of 1.5 in MMSE between the two study groups with 80% power and 5% probability of type 1 error, 29 subjects were required per group. This calculation assumed an SD of 2 in the MMSE score. Thus, 30 patients in each group were recruited. The exclusion criteria includedthose whose MMSE score at screening was ≤23, patients with diseases of the central nervous system, patients with history of consumption of tranquilizers or antidepressants, patients with severe visual or auditory handicap, patients currently diagnosed with alcoholism or drug dependence, history of previous neuropsychological testing, those having a geriatric depression score ≥21, patients undergoing a second procedure even if unrelated and patients of ASA grade III and IV (i.e., with coexisting severe cardiovascular, respiratory, renal or hepatic diseases).

The study protocol was approved by the Institutional Ethics Committee. All the patients or their caregivers were explained about the purpose of the study and were ensured strict confidentiality, and a written informed consent was obtained from each of the patients prior to the study. They were given the option of not participating in the study if they did not want to.

All patients were screened using MMSE. Patients with scores fulfilling the inclusion criteria were further tested with the components of the Kolkata Cognitive Screening Battery. Our cognitive screening test battery consisted of category-based verbal fluency tests (fruits and animals), a 15-item version of the object naming test, mental state examination, calculation tests, visuo-constructional ability (which included drawing the circle, diamond, overlapping rectangles and box) and a set of memory tests that consisted of immediate memory, delayed and recognition of a 10-item wordlist. This test battery has already been used and validated by Ganguli and her colleagues in a rural Ballabgarh population in north India and was named the Kolkata Cognitive Screening Battery.[19–21] Patients were subsequently randomized into two groups, groups A and B, to receive GA and EA, respectively, representing an equal number in both groups and an equal distribution of sexes. All the patients were pre-medicated with oral diazepam 5 mg 2 h prior to surgery. Patients were monitored with electrocardiography, pulse oximetry and non-invasive blood pressure measurement.

Patients of group A were pre-oxygenated for 3 min and administered fentanyl (2 μg/kg). Induction was achieved with thiopentone sodium (4–6 mg/kg) intravenously till loss of eye lash reflex was observed. Muscle relaxation was acquired with vecuronium bromide (100 μg/kg) and the participants were ventilated with face mask till cuffed endotracheal tube of appropriate size was inserted under direct laryngoscopic visualization. After intubation, its position was confirmed using capnography and chest auscultation and then mechanically ventilated. Patients were subsequently maintained on nitrous oxide (66%) in oxygen and halothane (0.5 MAC), with top-up doses of fentanyl and vecuronium as required. After completion of surgery, the anesthetics were discontinued and the patients were extubated following reversal of neuromuscular blockade with neostigmine (50 μg/kg) and glycopyrrolate (10 μg/kg). Post-operative analgesia was maintained with tramadol (2 mg/kg) intramuscularly on request along with diclofenac sodium (75 mg) intramuscularly twice-daily. In group B patients, under strict aseptic precaution and on infiltration with local anesthetic, a epidural 18G multiorifice catheter was placed 2 cm inside the lumbar epidural space through a 16G Tuohy epidural needle. The epidural space was identified by the loss of resistance technique using saline. A test dose of 3 ml of 2% lignocaine with 1:200000 adrenaline was administered through the epidural catheter port on negative aspiration of cerebrospinal fluid and blood. This was to rule out accidental intrathecal or intravascular placement. On confirmation of proper positioning within the epidural space, intraoperative anesthesia was induced with 16–18 ml of 0.5% bupivacaine. Subsequent top-up doses were given according to requirement and duration of surgery. In the post-operative period, analgesia was maintained with intermittent epidural boluses of 50 mg tramadol in 10 ml normal saline at 6-hourly intervals. Catheters were removed after 48 h following surgery. Surgeries were undertaken as a routine procedure and the operated patients were re-evaluated after 1 week using the Kolkata Cognitive Screening Battery as done pre-operatively.

The study findings were disseminated to the patients and their caregivers in health education sessions to complement the findings of the study.

### Statistical analysis

The collected data were thoroughly cleaned and entered into MS-Excel spread sheets and analysis was carried out. The procedures involved were transcription, preliminary data inspection, content analysis and interpretation. The statistical analyses were carried out using Graph Pad In Stat “version 3” software. Cognitive screening scores were compared between the two groups using Student’s unpaired *t*-test, with a Bonferroni correction for multiple comparisons.

## RESULTS

The two groups were comparable regarding age and sex. The mean age of the patients undergoing surgery under GA was 67.13 years (SD 7.10) while those operated under lumbar EA had a mean age of 66.63 years (SD 5.61). The two groups were also comparable with regard to the type of surgery being undertaken. A majority (30.00%) of the patients underwent bipolar prosthesis, followed by Austin Moore prosthesis (25.00%) and dynamic hip screw (21.6%) placement.[Table 1].

**Table 1:** Distribution of groups on the basis of type of surgery

Type of surgery	Groups		Total n (%)
	A	B
Austin Moore prosthesis	7	8	15(25.00)
Bipolar prosthesis	9	9	18 (30.00)
Dynamic hip screw	7	6	13 (21.67)
Cortical hip screw	1	0	01 (1.67)
Interlocking subtrochanteric fracture	1	0	01 (1.67)
Total knee replacement	2	4	06 (10.00)
Total hip replacement	3	3	06 (10.00)

GROUP A: PATIENTS UNDER GENERAL ANESTHESIA; GROUP B: PATIENTS UNDER EPIDURAL ANESTHESIA

The mean and SD of both groups were also comparable in terms of various cognitive parameters, as assessed pre-operatively. Group A had a mean of 28.96 in terms of verbal fluency, 14.86 in object naming test, 27.30 in MMSE, 12.03 in visual–constructional ability, 4.73 in calculation ability, 19.93 in immediate memory recall and 19.56 in delayed memory recall. Similarly, in group B, the means were 30.80, 14.90, 28.03, 12.36, 4.76, 20.93 and 19.53 in terms of verbal fluency, object naming test, MMSE, visual–constructional ability, calculation ability, immediate memory recall and delayed memory recall, respectively. The aggregate score in group A was 135.33 and in group B was 140.26. There was no significant statistical association between the two groups. Post-operatively, group A had a mean of 24.40 in terms of verbal fluency, 14.86 in object naming test, 25.16 in MMSE, 11.36 in visual–constructional ability, 4.33 in calculation ability, 21.96 in immediate memory recall and 19.43 in delayed memory recall. Similarly, in group B, the means were 27.86, 14.90, 26.83, 11.50, 4.30, 23.40 and 19.46 in terms of verbal fluency, object naming test, MMSE, visual–constructional ability, calculation ability, immediate memory recall and delayed memory recall, respectively. The aggregated score in group A was 125.00 and that in group B was 132.00. There was a significant statistical association between the two groups in regard to post-operative verbal fluency, MMSE, immediate and delayed memory recall and aggregate score. However, these differences were not significant after the application of the Bonferroni correction for multiple analyses, except the significant differences observed only in the MMSE scores. The two groups were not comparable in terms of object naming test, visual–constructional ability and calculation ability [Table 2].

**Table 2:** Post-operative cognitive function scores in response to general and epidural anesthesia

Parameters	Pre-operative			Post-operative		
	Mean	SD	*P*-value	Mean	SD	*P*-value
Verbal fluency						
Group A	28.96	6.27	t = 1.104, df 58; *P* = 0.1370	24.40	6.60	t = 2.082, df= 58; *P* = 0.0209
Group B	30.80	6.63		27.86	6.27
Object naming test						
Group A	14.86	0.34	t = 0.4832, df = 58; *P* = 0.3154	14.86	0.34	t = 0.4832, df = 58; *P* = 0.3154
Group B	14.90	0.30		14.90	0.30
Mini mental state examination						
Group A	27.30	1.84	t = 1.095, df = 58; *P* = 0.1390	25.16	2.76	t = 2.656, df = 58; *P* = 0.0051
Group B	28.03	1.27		26.83	2.06
Visuo–constructional ability						
Group A	12.03	1.37	t = 1.095, df = 58; *P* = 0.1390	11.36	1.99	t = 0.2908, df = 58; *P* = 0.3861
Group B	12.36	0.92		11.50	1.73
Calculation ability						
Group A	4.73	0.63	t = 0.1949, df = 58; *P* = 0.4231	4.33	1.44	t = 0.08441, df = 58; *P* = 0.4665
Group B	4.76	0.56		4.30	1.31
Memory: *Immediate recall*						
Group A	19.93	4.54	t = 0.9830, df = 58; *P* = 0.1648	21.96	3.22	t = 1.886, df = 58; *P* = 0.0322
Group B	20.93	3.23		23.40	2.67
Memory: *Delayed recognition*						
Group A	19.56	0.77	t = 0.1549, df = 58; *P* = 0.4387	19.43	1.94	t = 0.07576, df = 58; *P* = 0.4699
Group B	19.53	0.73		19.46	0.97
*Aggregate score*						
Group A	135.33	13.44	t = 1.574, df = 58; *P* = 0.0605	125.00	17.61	t = 1.764, df = 58; *P* = 0.0415
Group B	140.26	10.67		132.00	12.73

GROUP A: PATIENTS UNDER GENERAL ANESTHESIA; GROUP B: PATIENTS UNDER EPIDURAL ANESTHESIA

## DISCUSSION

We observed no statistical difference in cognitive behavior in either group pre-operatively who were comparable with respect to age, sex and type of surgery. A significant difference was observed between the two groups with respect to only the MMSE scores of the Kolkata Cognitive Screening Battery.

In the study conducted by Rasmussen *et al*., regional anesthesia (20/158; 12.7%) was associated with a significantly low incidence of POCD compared with GA (33/156; 21.2%) 1 week after surgery. However, the incidences declined at 3 months following surgery (13.9% vs. 14.3%) and were found to be statistically non-significant.[14] William-Russo *et al*., in a prospective, randomized study comparing two groups receiving EA and GA undergoing unilateral total knee replacement, got no significant differences post-operatively at 1 week and 6 months. Overall, 5% of the patients exhibited cognitive decline at 6 months post-operatively, but with no statistically significant difference between the two anesthesia groups.[22] A study by Bryson *et al*.[23] and Jones *et al*.[24] on elderly patients similarly did not demonstrate any difference in outcome regarding cognitive and functional competence between the two groups receiving regional or GA. Papaioannou *et al*. demonstrated significant differences between the general and the regional techniques with respect to cognitive outcome in elderly following 3 days of surgery.[25] Tzabar *et al*. in a similar study found a significant deterioration in ambulatory patients with GA in comparison to local anesthesia with respect to cognitive performance.[26]

The Alexandria University study compared the effect of GA or regional vertebral analgesia (subarachnoid or epidural) on post-operative cognitive function in patients undergoing orthopedic and urologic surgery. Using the Wechsler Adult Intelligence Scale revised pre-operatively and post-operatively, cognitive functions did not change significantly in young adult patients after either general or regional vertebral nor in elderly patients who received regional vertebral as compared with the pre-operative levels. Regional vertebral analgesia was observed to be advantageous over GA for elderly patients in terms of a better post-operative neuropsychological functioning.[27]

In the Peking University First Hospital study, cognitive function was assessed pre-operatively and 7 days post-operatively using a battery of nine neuropsychological tests in elderly patients. Early POCD occurred in 46.7% of the elderly patients undergoing abdominal surgery. However, there was no significant difference between the effects of the two different methods of anesthesia and post-operative analgesia on the incidence of POCD.[28] A study from France to evaluate the effects of anesthesia on the incidence of cognitive dysfunction after orthopedic surgery in elderly patients in a French post-operative cognitive decline persisted for up to 3 months in 56% of the subjects. Dysfunction was limited to verbal, visuo-spatial and semantic abilities and secondary and implicit memory.[29]

Machado *et al*. evaluated the cognitive function of patients (ASA grade I and II) submitted to orthopedic, urologic, gynecologic, general and ENT surgery using the mini mental state test, which was performed 24 h before and 24 h after anesthesia. There was a high correlation between pre- and post-operative MMS scores (R = 0.94), which was comparable with our study. Patients in the general balanced anesthesia group showed a significant (*P* < 0.001) decrease in cognitive performance 24 h after surgery in comparison with the pre-operative test. No differences were observed between the pre- and post-operative MMS tests in the total intravenous anesthesia and regional anesthesia groups.[30]

### Strength of the study

Early POCD was seen following GA or EA in elderly subjects undergoing hip and knee surgery, and their comparative effects will be considered as modus operandi of anesthesia to evolve a system for holistic care in its positive influence on the process of recovery.

#### Limitations of the study

It could have been better if we could have followed the post-operative cases at 3 and 6 months respectively to delineate the prognosis of the dysfunctions because early POCD is largely reversible when followed over a period. Also, as there are many risk factors for POCD and all of them were not taken care of, the results may be confounding. It would have been better if we could have extended our study to incorporate social factors related to the research. Despite this limitation, results are believed to be significant, considering that significant number of patients undergoing only orthopedic interventions were recruited in comparison to studies reported in previous literatures.

### Future directions of the study

The target organ for anesthetic drugs is the central nervous system. For years together, we had reason to believe that their effects do not outlive their pharmacological actions and that the function of the brain is restored once the drug gets eliminated. There is a need for compilation of increasing evidence in this direction.

## CONCLUSIONS

Our study showed a significant statistical difference in cognitive functions in patients undergoing hip and knee surgery following GA or EA. Despite several attempts, no gold standard treatment has been devised for POCD. Numerous techniques have been proposed to minimize the risk and reduce perioperative cerebral damage. Such suggestions are rarely evidence based and merely empirically developed strategies. The main objective is to maintain a stable hemodynamic and physiological state, including ventilation, metabolism and blood chemistry. Hypoxia, hypo or hypercapnoea, hypo or hyperglycemia and alteration of sodium amplify the clinical impact of acute vascular cerebral damage and thus their prevention might have a role in reducing the incidence of POCD.
